# Getting to the Point: A Community-Designed, Low-Barrier Hepatitis C Testing and Treatment Program for People Who Inject Drugs in Rural America

**DOI:** 10.3390/v17121589

**Published:** 2025-12-06

**Authors:** Amanda N. Perry, Elizabeth Eccles, Shoshana H. Bardach, Alastair Huntley, Ryan Fowler, David de Gijsel

**Affiliations:** 1 The Dartmouth Institute for Health Policy and Clinical Practice, Geisel School of Medicine at Dartmouth, Lebanon, NH 03756, USA; amanda.n.perry@dartmouth.edu (A.N.P.);; 2 Dartmouth Health, Section of Infectious Diseases and International Health, Lebanon, NH 03756, USA; 3 The HIV/HCV Resource Center, Lebanon, NH 03766, USA

**Keywords:** hepatitis C, HCV, HCV treatment, syringe service program, point-of-care testing, linkage to care, people who inject drugs, PWID

## Abstract

Background: People who inject drugs (PWID) have a higher risk of contracting hepatitis C (HCV) than the general population, but these individuals are often poorly served by traditional healthcare systems. The elimination of HCV as a threat to public health relies on the treatment of this population. Novel care models designed with input from PWID may help to better align care to the needs of PWID. Methods: We designed and implemented a community-based, point-of-care testing program for HCV delivered by a syringe service program, combined with facilitated access to a healthcare provider, care navigation, and financial incentives. We collected participant demographics and drug use patterns, testing and treatment history, and communication preferences. Descriptive analyses include the number of people tested between 15 October 2021 and 1 February 2025, their seropositivity rate, and the number who completed pre-treatment laboratory tests, completed treatment and achieved cure by sustained virologic response (SVR12) by 1 August 2025. Results: The program engaged 464 unique individuals, of whom 98 (21.1%) had a known diagnosis of HCV. Of 389 unique individuals who underwent point of care (POC) HCV antibody (Ab) testing, including 31 with a known prior diagnosis of HCV, 97 (24.9%) had a positive result. Of 439 unique individuals who underwent POC HIV Ab testing, only 1 had a positive result. Of 164 individuals with either a positive POC HCV Ab test or a known HCV diagnosis, 66 completed pre-treatment laboratory tests, identifying 52 viremic participants. Of those, 35 started and completed treatment. Among those who completed treatment, 9 (25.7%) achieved SVR12, 3 (8.6) failed to achieve SVR12, and 23 (65.7%) had outstanding laboratory orders for SVR12 determination. Conclusions: An incentivized, community-based, point-of-care testing program with facilitated linkage to care successfully engaged a high-risk population in HCV and HIV testing and treatment. However, substantial attrition occurred at each step of the care cascade, particularly at SVR12 determination. Additional strategies are needed to optimize retention throughout the entire care cascade.

## 1. Intro/Background

Estimates suggest that about 4 million people in the US are infected with hepatitis C (HCV) and nearly half of those do not know their status [1]. Further, worldwide, approximately 38.8% of people who inject drugs (PWID) have a current HCV infection [2]. HCV is preventable with the right precautions and is now curable, but HCV elimination requires increased uptake of harm reduction strategies and treatment. The World Health Organization’s global hepatitis strategy aims to reduce new hepatitis C infections by 90% between 2016 and 2030 [3].

Despite public health elimination efforts and treatment options with limited side effects, PWID are not routinely accessing HCV treatment, likely due to limited awareness of HCV treatment options, competing life priorities (e.g., securing food, housing), concerns about side effects, and fear of being stigmatized [4]. PWID also face challenges when engaging in healthcare, including traumatization in the medical setting and difficulty navigating the systems that surround healthcare in the United States [5]. These barriers can be exacerbated in rural settings, areas of the US that are heavily affected by opioid use [6]. The challenges and negative experiences PWID have while interacting with the U.S. health system cause PWID to withdraw from mainstream healthcare systems, making engagement in treatment for conditions like HCV more unlikely [5].

Through a quality improvement initiative at a large, academic medical center in rural, northern New England, USA, a multidisciplinary team developed a community-based, HCV point-of-care testing program through a local syringe service program (SSP), which included incentivized and facilitated linkage to treatment [7]. Prior research suggests that PWID interact with SSPs far more frequently than other healthcare settings [8]. The program was designed such that a trusted individual in the community of PWID (e.g., SSP workers) would be able to vouch for a medical provider. This medical provider offered treatment using a trauma-informed approach, without stigma, and with respect. The target population lived in rural, west-central New Hampshire and rural, east-central Vermont. Importantly, the program was designed with individuals with lived experience and developed in response to the local community needs [1]. The multidisciplinary group that developed the program included 2 team members and an 8-person Community Advisory Board, all with lived experience using drugs. Additional details about the development of the program can be found in a previous publication by Bardach and colleagues [7]. This manuscript evaluates the outcomes of the first four years of this testing program, from 15 October 2021 through 1 August 2025, and describes participants’ activity through the care cascade for HCV.

## 2. Methods

The program called “To The Point,” or TTP, trained employees at a local SSP and a nurse from a local health center to offer rapid, point-of-care (POC) HCV and HIV antibody (Ab) testing (OraQuick by OraSure Technologies, Bethlehem, PA, USA) on fingerstick capillary blood samples to SSP participants and the participants’ contacts. Eligibility criteria included: adults ≥ 18 years of age who were either current or former users of injection or non-injection drugs, or close contacts of people who use drugs. Recruitment occurred primarily at local community resource centers, through SSP outreach, and at community events.

The TTP team consisted of a doctor (MD), a nurse (RN), a program manager, an SSP worker, and a medical assistant. Collectively, this team provided outreach, care coordination, health education, and treatment for HCV. The program aimed for weekly contact with participants following testing and during the treatment phase, with text messaging as the primary mode based on participant preference. Contact frequency was tailored to individual participant needs and preferences, ranging from daily to monthly check-ins.

Participants who were already aware that they were infected with HCV were offered confirmatory laboratory-based HCV Ab and reflex RNA testing without a POC HCV Ab test. Before testing, participants completed an optional paper survey that collected demographic data, risk factors including a history of injection drug use and sex work, history of prior testing and treatment, barriers to care, and preferences for future contact by the TTP team. Paper surveys were then entered and stored by program staff in a REDCap database.

**Figure 1:**
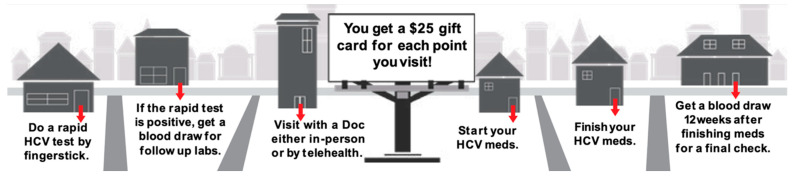
TTP Program Treatment Process for Hepatitis C. Gift cards were cash cards and could be used anywhere that accepted credit/debit, providing similar flexibility as cash while avoiding the need for staff to have large amounts of cash on hand. When the participant was met in-person, gift cards were handed to the individual; when the participant was not in person, gift cards were mailed to the address of their choosing, or were available for pickup at the SSP.

The SSP operated ‘on demand’ without a fixed site, meeting clients in the community or at their home (i.e., street testing). HCV testing was offered in conjunction with the delivery of injection equipment, or as a stand-alone service. If a participant tested positive for HCV Ab out in the community, the person conducting the testing provided counseling, harm reduction information, and offered a connection to a member of the healthcare team (free of charge). The participant was given a pocket card that showed next steps in the HCV clearance cascade (See Figure 1), the financial incentives available at each step, and contact information for the program. If the participant agreed to be contacted, the nurse followed up, navigated, and coordinated steps along the clearance cascade with the participant.

TTP was conceptualized to street test individuals out in the community, at their homes, and at SSP meet-up locations; however, based on feedback and requests, and to facilitate initial connection to a healthcare provider, the program expanded and held testing events at local community resource centers, with healthcare providers present for consultations. SSP workers were present at all testing events and used their connections to increase awareness for, and trust in, the testing events and the clinical providers. If the participant chose to connect with a member of the healthcare team, the healthcare provider (RN or MD) offered further counseling on the test results, education about HCV, explanation of next steps towards HCV treatment, assessment of other immediate health needs, evaluation of insurance status, and set up a future healthcare appointment. Confirmatory and HCV treatment laboratory tests were ordered through the laboratory of the participant’s choice. Additionally, about 18 months into the program, a medical assistant intermittently joined the team and performed venipuncture in the community to collect HCV treatment laboratory tests, removing the need for a participant to make a subsequent trip to a fixed phlebotomy site. Unfortunately, the program could not assure a consistent presence of the phlebotomist, precluding an analysis of their impact on outcomes.

Once the confirmatory laboratory results were received by the care team, any participant with active HCV infection was offered a follow-up appointment with an MD—usually within 3 weeks. Fibrosis assessments were performed based on a FIB4 score. A FIB4 score > 3.25 was considered indicative of cirrhosis and prompted another blood draw for a prothrombin time (INR) to determine the Child-Turcotte-Pugh (CTP) classification of cirrhosis severity. Full hepatitis B and HIV serologies were obtained but HCV genotype was only checked if required by the participant’s insurance. Any treatment-naïve participant with a FIB4 score < 3.25 or with a FIB4 score > 3.25 and a CTP score < 7 with negative hepatitis B surface antigen (HBsAg) were offered treatment according to the simplified treatment algorithm of the American Association for the Study of Liver Diseases and the Infectious Diseases Society of America (AASLD/IDSA) HCV Guidance with glecaprevir/pibrentasvir for 8 weeks or sofosbuvir/velpatasvir for 12 weeks [9]. No on-treatment monitoring was performed and cure was established by an undetectable HCV RNA obtained at least 12 weeks after treatment completion (SVR12). A specialty pharmacy dispensed the medication and shipped them either to the participant or to the SSP for pickup by or delivery to the participant, unless otherwise directed by the insurance provider.

The Dartmouth Health Institutional Review Board determined that the project did not require review and approval because the proposed activities did not meet the definition of research involving human subjects as defined by federal Department of Health and Human Services regulations.

### Analysis

To allow for anonymous testing and to account for the sometimes hurried context of mobile syringe programs, participants could choose not to provide key data such as their demographic information or prior testing history, resulting in incomplete data sets for many participants. Rather than restrict the analysis to participants with complete data, variables are reported as ‘not answered’ when a participant was not asked or did not answer a particular item. Descriptive statistics were used to characterize the population served, contact preferences, and prior testing experiences and results. Duplicate test results were included in this analysis but for individual participant data, duplicates were consolidated. The TTP HCV clearance cascade only includes unique individuals who were found to be HCV seropositive before 1 February 2025, to allow for sufficient time (24 weeks) to have potentially completed treatment and be eligible for SVR12 determination. Treatment and follow up laboratory data were collected through 31 July 2025.

## 3. Results

Between 15 October 2021 and 1 February 2025, 464 unique individuals engaged with the program. Most participants were engaged at community testing events held at local community resource centers. Just over half of individuals tested were male (52.8%), and the majority were White, non-Hispanic, and cisgender. At time of first test, the median age was 39.6 years, ranging from 19 years to 86 years. Of 193 (41.6%) participants who answered questions about their drug use history, 86 (18.5%) reported injecting within the past 5 years.

In terms of contact preferences, a plurality of participants preferred communicating by text message (37.9%), followed by phone call (20.9%), and only a small subset (6.5%) preferred email (See Table 1).

At the time of first test, 123 (26.5%) participants had never been tested for HIV or HCV before. Ninety-eight (21.1%) individuals knew that they had a prior positive HCV test, representing 33.9% of the 289 people who ever had a HCV test (the survey did not distinguish between HCV Ab or RNA positivity, but the vast majority of these tests likely were HCV Ab tests). 25 (5.4%) reported prior HCV treatment, or 25.5% of the 98 people with a positive HCV test (See Table 2).

### Test Results and Clearance Cascade

A total of 606 POC HIV Ab tests yielded 1 newly positive and 605 negative results among 439 unique individuals. The person with the positive result already knew that he was also HCV infected and was successfully linked to care and started on antiretroviral treatment as well as DAAs. Of 510 POC HCV Ab tests among 389 unique individuals, 110 were positive and 400 negative. The 110 positive results occurred in 97 unique individuals as 9 people were tested twice, and 2 people thrice despite a known prior positive result. Thus, the HCV seropositivity rate among the 389 tested participants was 97/389 or 24.9%.

In addition, of the 98 individuals with known prior HCV, 67 did not undergo HCV Ab testing but were directly referred to a laboratory for follow up testing. The remaining 31 participants did undergo HCV Ab testing in violation of the program’s protocol and are included in the tally of unique participants above.

Among the combined 164 individuals eligible for the care cascade (97 who tested positive for HCV Ab + 67 with reported prior HCV infection), 66 (40.2%) completed RNA testing, identifying 52 (78.8%) viremic participants.

**Figure 2:**
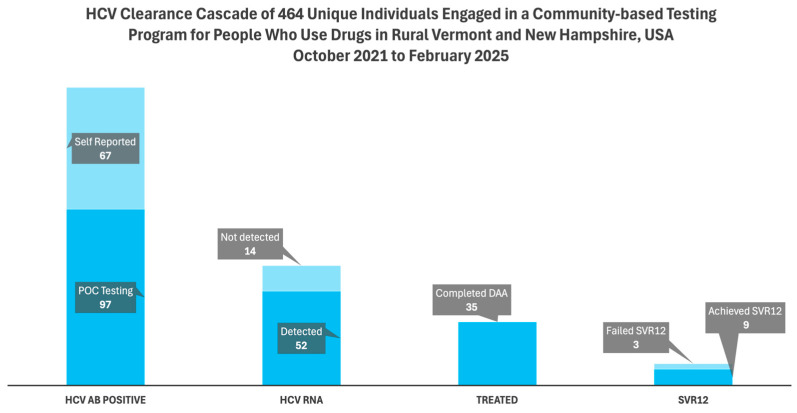
TTP HCV Clearance Cascade. Step-offs illustrate the loss to follow up or delay in re-engagement of participants to achieve the next step in the workup, treatment and determination of cure of active hepatitis C. POC = point of care; DAA = direct acting antiviral; SVR12 = sustained virologic response 12 weeks post treatment.

Of the 52 viremic participants, 37 (71.2%) had documented follow up with the project team, while 35 (67.3%) started and completed treatment with DAAs. Of the 35 who completed treatment, 9 (25.7%) achieved documented SVR12, 3 (8.6%) failed to achieve SVR12, and the remaining 23 (65.7%) had outstanding orders for SVR12 labs as of 1 August 2025 (See Figure 2).

## 4. Discussion

The To The Point project successfully engaged communities of PWID in rural New England and facilitated HCV testing and treatment through an innovative, community-based approach. Despite the recognized importance of treating PWID to achieve HCV elimination goals and to improve individual health outcomes, this population remains underserved [10,11]. The project’s co-design with healthcare professionals and PWID, the integration with an SSP, and the flexible options to engage in follow up care were informed by studies evaluating factors that improve engagement and cure among PWID [12,13].

Our results demonstrate both successes and challenges in HCV care delivery to PWID. The 40.2% RNA testing completion rate and 67.3% treatment initiation among viremic patients are comparable to other facilitated HCV programs dedicated to PWID [14]. However, these outcomes reveal substantial room for improvement, particularly when compared to programs offering same-day RNA testing or reflex testing protocols, which have achieved RNA testing rates exceeding 75% [15,16]. The low SVR12 documentation rate (34.3% of those completing treatment) represents a critical gap in confirming cure. This finding aligns with other community-based programs where post-treatment follow-up remains a challenge [17]. While the actual cure rate may be higher given the efficacy of DAAs, the inability to document cure limits program evaluation and participant reassurance

Several factors contributed to the program’s successes. The community presence and collaboration with a local SSP likely increased trust among the participants and decreased perceived stigma, improving the odds of accessing follow up care. Offering participants the option to have confirmatory laboratory tests performed at any laboratory of choice and, when available, directly upon learning of a positive HCV Ab through on-site phlebotomy demonstrated respect for participant autonomy and reduced logistical barriers to confirmatory testing. Prior research suggests that enhancing patient autonomy can increase motivation for health management behaviors [18].

Persistent follow up by our nurse and the SSP partners via preferred means of communication, which often meant text messaging, strengthened relationships with participants. Rather than considering participants “lost to follow up” after a certain period, the program maintained an open-ended invitation to access care, with laboratory orders that remained valid for one year.

Visits with the medical provider were offered in various formats to decrease barriers: telehealth using video or audio-only connections, brief visits at testing events, and facilitated telehealth visits in the community through tablets carried by SSP staff. The relatively low immediate uptake of medical provider consultations (approximately 15% of those testing positive) likely reflects the competing priorities and time constraints faced by PWID, particularly those actively using substances. Medication delivery and adherence support was facilitated by a mission-driven specialty pharmacy and a dedicated TTP nurse, likely contributing to the high treatment completion rate among those who initiated therapy.

Despite program innovations, significant attrition occurred at each step of the care cascade. The gap between antibody positivity and RNA testing (59.8% lost) represents the largest single drop-off point. Point-of-care RNA testing, though more expensive, could eliminate this barrier entirely. Programs using GeneXpert or similar technologies have demonstrated near-complete RNA testing rates [19,20].

The inability to confirm cure through SVR12 testing remains problematic. Participants may assume cure based on treatment completion, or the three-month delay between treatment completion and SVR12 eligibility may prove insurmountable. Strategies to improve SVR12 documentation might include increased financial incentives, interim check-ins during the waiting period, SVR4 as a proxy measure, or alternative testing methods such as dried blood spot collection [21].

The project has several limitations. First, the program reached mostly white, cisgender individuals, with limited diversity in race/ethnicity. The proportion of participants reporting injection drug use (18.5%) was lower than anticipated for a program operating through an SSP, suggesting that the expansion to community resource centers may have diluted the focus on the highest-risk population. However, due to the high rate of non-response to questions about drug use (58.4%), the true rate of injection drug use was likely much higher.

Second, to reduce intrusiveness and maintain low barriers to engagement, TTP collected limited data from participants. This restricted our ability to analyze factors associated with progression through the care cascade, identify specific barriers, and tailor interventions accordingly.

Third, the role of financial incentives warrants further study. Several participants explicitly shared that they agreed to initial testing solely for the financial reward without intention to follow up. While knowing one’s HCV status has value, testing without treatment intention limits achievable cure rates.

Finally, the study design did not include a control group, limiting our ability to quantify the program’s impact compared to standard care. However, given the well-documented poor outcomes of PWID in traditional healthcare settings, the ethical considerations of withholding this intervention would be substantial.

In summary, the TTP project demonstrated that community-based, peer-supported HCV testing and treatment programs can successfully engage PWID in rural settings. The program achieved excellent rates of testing uptake and reasonable rates of treatment initiation. However, substantial attrition at each cascade step, particularly RNA testing and SVR12 documentation, highlights the need for continued innovation. Future efforts should focus on community partnership, co-creation of healthcare delivery with people with lived experience and the impact of trust, stigma-free care, flexibility and extended timelines before considering someone lost to care. Further decreasing the number and complexities of steps in the clearance cascade, such as full point-of-care testing including HCV RNA and HBV serologies and increasing care navigation and peer support would likely increase the rate of cure among PWID.

## Figures and Tables

**Table 1:** TTP Participant Demographics, Contact Preferences and Drug Use History (n = 464).

**Age (at Time of First TTP Test, if Tested Multiple Times)**	**Years, n (%)**
Median	39.6
Range	19–86
Not answered	60 (12.9%)
**Gender (unique individuals)**	**n (%)**
Male	245 (52.8%)
Female	182 (39.2%)
Transgender	1 (0.2%)
Non-Binary	5 (1.1%)
Not answered	31 (6.7%)
**Race**	**n (%)**
White	359 (77.4%)
Black or African American	9 (1.9%)
American Indian/Alaskan Native	8 (1.7%)
Other	13 (2.8%)
Not answered	75 (16.2%)
**Hispanic or Latino**	**n (%)**
No	306 (65.9%)
Yes	22 (4.7%)
Not answered	136 (29.3%)
**Preferred method of contact**	**n (%)**
Phone Text	176 (37.9%)
Phone Voice	97 (20.9%)
Email	30 (6.5%)
Other (e.g., friend or family member)	14 (3.0%)
Not answered	147 (31.7%)
**Injected drugs in the past 5 years**	**n (%)**
Yes	86 (18.5%)
No	107 (23.1%)
Not answered	271 (58.4%)

**Table 2:** TTP Participant Prior Testing and Results (n = 464).

**Prior Testing**	**n (%)**
Yes, Both HCV and HIV	272 (58.6%)
Yes, HCV Only	17 (3.7%)
Yes, HIV Only	31 (6.7%)
No, Neither HCV nor HIV	123 (26.5%)
Not sure	7 (1.5%)
Not answered	14 (3.0%)
**Prior HCV Test Results**	**n (%)**
Positive	98 (21.1%)
Negative	168 (36.2%)
Unsure	19 (4.1%)
Not applicable (no prior test)	179 (38.6%)
**Prior HCV Treatment**	**n (%)**
Previously treated	25 (5.4%)
Not previously treated	71 (15.3%)
Not applicable (no prior positive test)	366 (78.9%)
Not answered	2 (0.4%)
**Unique HCV Ab test results**	**n (%)**
Positive	97 (24.9%)
Negative	292 (75.1%)
Not tested	75

## Data Availability

The raw data supporting the conclusions of this article will be made available by the authors on request.
